# Nanotechnology Approaches for the Remediation of Agricultural Polluted Soils

**DOI:** 10.1021/acsomega.3c09776

**Published:** 2024-03-13

**Authors:** Anand Raj Dhanapal, Muthu Thiruvengadam, Jayavarshini Vairavanathan, Baskar Venkidasamy, Maheswaran Easwaran, Mansour Ghorbanpour

**Affiliations:** † Chemistry and Bioprospecting Division, Institute of Forest Genetics and Tree Breeding (IFGTB), Forest Campus, Indian Council of Forestry Research and Education (ICFRE), Coimbatore 641 002, Tamil Nadu, India; ‡ Department of Crop Science, College of Sanghuh Life Science, Konkuk University, Seoul 05029, Republic of Korea; § Department of Biotechnology, Karpagam Academy of Higher Education, Coimbatore 641 021, Tamil Nadu, India; ∥ Department of Oral & Maxillofacial Surgery, Saveetha Dental College and Hospitals, Saveetha Institute of Medical and Technical Sciences (SIMATS), Saveetha University, Chennai 600 077, Tamil Nadu, India; ⊥ Department of Research Analytics, Saveetha Dental College and Hospitals, Saveetha Institute of Medical and Technical Sciences (SIMATS), Saveetha University, Chennai 600 077, Tamil Nadu, India; # Department of Medicinal Plants, Faculty of Agriculture and Natural Resources, Arak University, Arak 38156-8-8349, Iran; ¶ Institute of Nanoscience and Nanotechnology, Arak University, Arak 38156-8-8349, Iran; ∞ Center for Global Health Research, Saveetha Medical College, Saveetha Institute of Medical and Technical Sciences (SIMATS), Saveetha University, Chennai, 600077, India

## Abstract

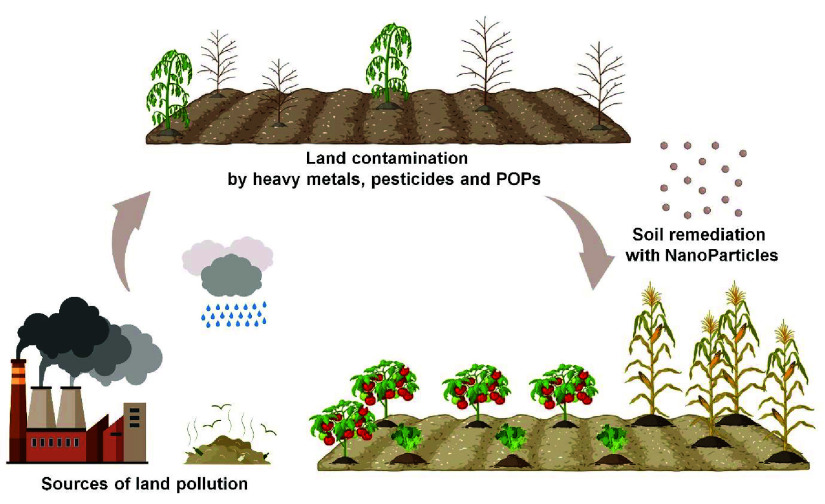

Soil pollution from various anthropogenic and natural activities poses a significant threat to the environment and human health. This study explored the sources and types of soil pollution and emphasized the need for innovative remediation approaches. Nanotechnology, including the use of nanoparticles, is a promising approach for remediation. Diverse types of nanomaterials, including nanobiosorbents and nanobiosurfactants, have shown great potential in soil remediation processes. Nanotechnology approaches to soil pollution remediation are multifaceted. Reduction reactions and immobilization techniques demonstrate the versatility of nanomaterials in mitigating soil pollution. Nanomicrobial-based bioremediation further enhances the efficiency of pollutant degradation in agricultural soils. A literature-based screening was conducted using different search engines, including PubMed, Web of Science, and Google Scholar, from 2010 to 2023. Keywords such as “soil pollution, nanotechnology, nanoremediation, heavy metal remediation, soil remediation” and combinations of these were used. The remediation of heavy metals using nanotechnology has demonstrated promising results and offers an eco-friendly and sustainable solution to address this critical issue. Nanobioremediation is a robust strategy for combatting organic contamination in soils, including pesticides and herbicides. The use of nanophytoremediation, in which nanomaterials assist plants in extracting and detoxifying pollutants, represents a cutting-edge and environmentally friendly approach for tackling soil pollution.

## 1 Introduction

**Figure 1:**
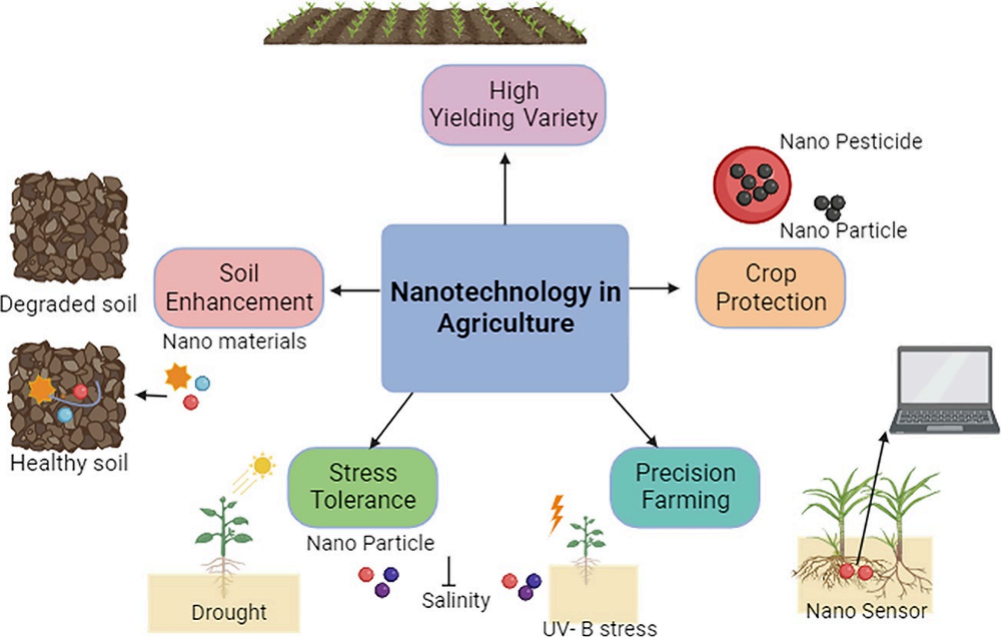
Nanotechnology approaches in agriculture.

Current agricultural practices have environmental consequences, even though they contribute significantly to meeting the ever-growing global demand for food. The expansion of agricultural methodologies has resulted in pervasive soil pollution with a diverse array of detrimental substances, such as pesticides and heavy metals.^1,2^ These contaminants pose significant threats to ecosystems and human health and compromise the fertility of agricultural land through the food chain.^3^ Given the aforementioned obstacles, it is crucial to investigate novel and environmentally sound remediation approaches that not only alleviate the consequences of soil contamination but also facilitate the restoration of soil vitality. The incorporation of nanotechnology into the agricultural sector represents a substantial advancement in tackling the complex issues that plague the worldwide food production system.^4^ The wide-ranging utilization of nanomaterials in precision agriculture, nutrient management, pest control, and soil health presents unparalleled prospects for the implementation of sustainable and effective farming methodologies^5^ (Figure 1). Recently, there has been a surge in the recognition of nanotechnology as a viable and groundbreaking approach to tackle the complexities linked to soil contamination in agricultural environments.^6^ The distinctive attributes exhibited by nanomaterials, including their substantial surface area, reactive nature, and ability to modify physicochemical properties, render them highly suitable for implementation in soil remediation.^6^ Nanotechnology has the capacity to fundamentally transform conventional methods of soil remediation through the provision of more effective, precise, and environmentally sustainable techniques to alleviate the consequences of agricultural contamination.^7^ The objective of this comprehensive review is to examine and consolidate the existing body of knowledge on the application of nanotechnology for the remediation of agriculturally contaminated soil. By conducting a comprehensive analysis of recent developments and research, we will explore a diverse array of nanomaterials, nanocomposites, and nanotechnological approaches utilized to eliminate, confine, and counteract soil impurities. Furthermore, the efficacy, environmental ramifications, and potential hazards linked to the utilization of nanotechnology for agricultural soil remediation are critically evaluated in this review. This review explores the revolutionary potential of nanotechnology for the remediation of soil contaminated by agriculture. Nanotechnology encompasses the intentional manipulation of substances at the nanoscale level and presents unparalleled prospects for the development and execution of customized remedies for soil remediation.

## 2 Soil Pollution Sources and Types

Soil is an intricate and vital ecosystem that provides a wide range of essential ecosystem services, including provisioning (e.g., freshwater, timber, food, and fiber), regulation (e.g., climate control, erosion prevention, and flood mitigation), cultural (e.g., aesthetic and spiritual values), and supporting (e.g., physical support for plants, animals, and human infrastructure) services.^8^ Soil health is defined as “the ability of soil to function as a dynamic living system within the limits of an ecosystem and land-use practices, supporting plant and animal productivity, enhancing water and air quality, and promoting overall plant and animal well-being”.^9^ Soil is a constantly evolving natural resource that consists of diverse elements, including gases, minerals, salts, organic and inorganic matter, and living organisms. It possesses biological, chemical, and physical characteristics that are sensitive to alterations, which can result from natural processes (such as volcanic eruptions, ore weathering, and forest fires) or, more frequently, from various human activities (disposal of household and industrial refuse and application of chemical fertilizers and pesticides to improve crop yields).^9^

Chemicals produced by humans and changes in the environment that occur naturally in the soil are the main causes of soil pollution. Soil contamination typically results from the breakdown of subterranean storage linkages, the use of pesticides, the seepage of polluted surface water into subsurface strata, the disposal of oil and fuel, the leaching of wastes from landfills, or the direct release of industrial wastes into the soil.^10^ The environment is continuously exposed to a variety of hazardous chemical components from both natural and anthropogenic sources. This is one of the many factors contributing to environmental contamination.^11^ Numerous parts of the world have become contaminated as a result of industrialization and urbanization because harmful substances are released from man-made sources. Sources of soil pollution are agricultural sources and nonagricultural sources such as industrial wastes, mining, and smelting.^11^

Soil ecosystem characteristics, including skeletal nature, depth, structure, humus content, nutrient availability, reactivity, foreign material presence, and edaphon, significantly impact production, buffering, filtering, and other soil functions.^12^ Soil quality cannot be assessed immediately but must be assessed through analyzing its properties.^12^ It is more accurate to consider physical, chemical, biological, and biochemical characteristics that are affected by environmental changes and land management. Physical characteristics include temperature, porosity, bulk density, and water holding capacity, while chemical parameters include reaction, carbon and nitrogen content, and nutrient content.^12^ Microbial factors seem to be particularly helpful in tracking heavy metal contamination of soil, although it is possible to quantify soil enzymes, respiration, C and N mineralization, biological N_2_ fixation, and the overall biomass of soil microorganisms.^13^ However, earlier studies have laid the foundation for understanding the traditional sources and types of soil pollution, and newer dimensions emphasize the interconnectedness of soil health with climate change, microbial ecology, sustainable practices, and advanced technologies. These insights are pivotal for crafting holistic strategies to address the evolving challenges of soil pollution and ensure a more resilient and sustainable environment.

## 3 Synthesis of Nanomaterials: Physical, Chemical, and Biological Methods

**Figure 2:**
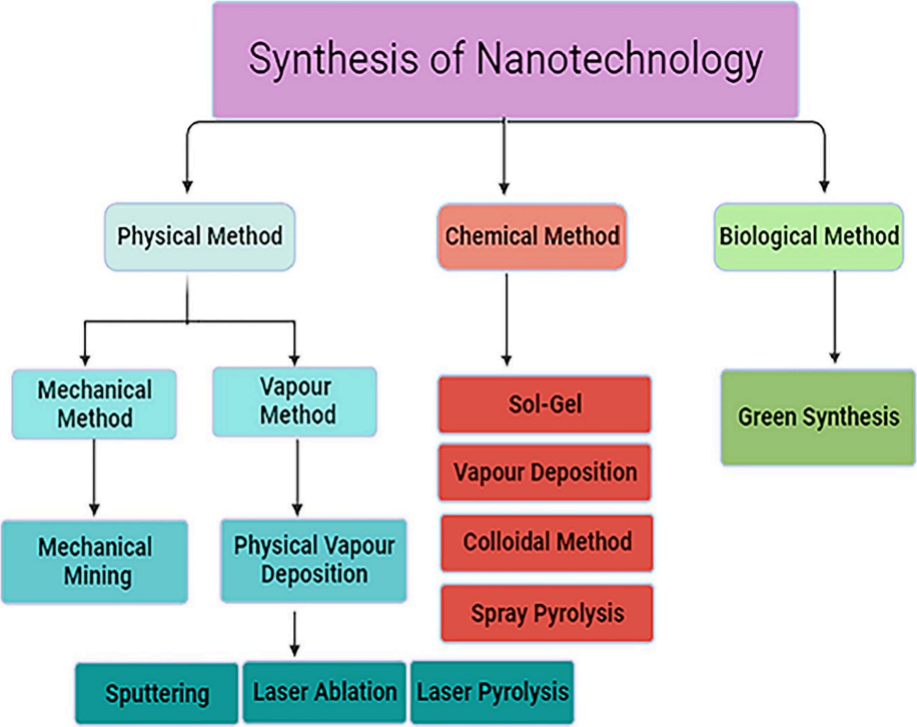
Synthesis of nanotechnology using various methods.

Nanoparticles have been generated through physical techniques, leveraging thermal energy, high-energy radiation, and mechanical pressure to induce material condensation, evaporation, abrasion, or melting.^14^ Physical approaches surpass chemical methods by ensuring the absence of solvent contamination in thin films and a uniform distribution of nanoparticles. These methods follow a top-down strategy, eliminating the need for solvents and consistently producing monodisperse nanoparticles. The commonly employed physical methods for nanoparticle synthesis include laser ablation, laser pyrolysis, physical vapor deposition, high-energy ball milling, and inert gas condensation.^15^ Nanoparticles can be produced through two commonly utilized methods: the top-down approach and the bottom-up approach.^16^ Different approaches have been used to create nanomaterials, including mechanical milling, sputtering, laser pyrolysis, laser ablation, electron beam evaporation, and nanolithography.^17^ This paragraph explores the diverse physical techniques for nanoparticle synthesis. Figure 2 depicts the nanoparticle synthesis using physical, chemical, and biological methods.

### 3.1 Physical Methods

#### 3.1.1 Mechanical Milling

Mechanical milling is a cost-efficient technique for generating nanoscale materials from large bulk materials.^18^ This method was effective in creating blends of diverse phases and played a pivotal role in crafting nanocomposites.^18^ The fundamental concept underlying the ball-milling method is critical to this process. Mechanical milling has been applied in the production of aluminum alloys fortified with oxides and carbides, as well as in the fabrication of wear-resistant spray coatings, nanoalloys based on aluminum/nickel/magnesium/copper, and various other nanocomposite materials. Ball-milled carbon nanomaterials have emerged as an innovative category of nanomaterials that offer opportunities to address environmental remediation, energy storage, and energy conversion needs.^18^

#### 3.1.2 Sputtering

Sputtering is a proficient technique for creating nanomaterials by bombarding solid surfaces with high-energy particles such as plasma or gas.^19^ During the sputtering deposition process, energetic gaseous ions bombard the target surface, leading to the expulsion of small atom clusters based on the incident gaseous-ion energy.^19^ This process, performed using a magnetron, radio frequency diode, and DC diode sputtering, typically occurs in a vacuum chamber with the introduction of sputtering gas.^19^ Subjecting the cathode target to a high voltage initiates collisions between the free electrons and gas, producing gas ions. These positively charged ions accelerate vigorously toward the cathode target, continually striking it and ejecting atoms from the target surface. For instance, magnetron sputtering has been employed for the crafting of WSe_2_-layered nanofilms on SiO_2_ and carbon paper substrates.^20^ This technique is compelling, as the composition of the sputtered nanomaterial mirrors the target material with fewer impurities, offering a cost-effective alternative to electron-beam lithography.^19,20^

#### 3.1.3 Laser Ablation

Laser ablation synthesis creates nanoparticles by directing a powerful laser beam onto the target material, causing the source material to vaporize because of the laser’s high energy, resulting in nanoparticle formation.^21^ Laser ablation for noble metal nanoparticle generation is environmentally friendly and eliminates the need for stabilizing agents or other chemicals.^21^ This method enables the production of various nanomaterials such as metal nanoparticles, carbon nanomaterials, oxide composites, and ceramics. Pulsed laser ablation in liquids is an intriguing approach for generating uniform colloidal nanoparticle solutions without surfactants or ligands. Nanoparticle properties, including average size and distribution, can be fine-tuned by adjusting the fluence and wavelength and introducing a laser salt. The sizes of the Pd nanoparticles synthesized in this manner are significantly influenced by the wavelength and fluence of the pulsed laser.^22^

### 3.2 Chemical Methods

#### 3.2.1 Sol–Gel

The sol–gel method comprises five main steps: hydrolysis, polycondensation, aging, drying, and thermal decomposition.^23^ During hydrolysis, the metal precursors undergo hydrolysis using either water (aqueous) or organic solvents (nonaqueous). Polycondensation involves condensation of neighboring molecules, removal of water or alcohol, and the formation of metal oxides. The aging process causes structural changes due to ongoing condensation.^23^ Drying, achieved through methods such as freeze-drying, thermal drying, or supercritical drying, leads to the creation of diverse structures such as aerogels and cryogels. The final step involved heat treatment, which eliminated the remaining water or alcohol molecules and other residuals. This step crucially controls the ultimate density of the material, making the heat-treatment temperature a pivotal parameter for regulation.^23^

#### 3.2.2 Chemical Vapor Deposition

Chemical vapor deposition (CVD) is a crucial process for the synthesis of carbon-based nanomaterials.^24^ This involves the formation of a thin film on the substrate surface through the chemical reaction of vapor-phase precursors. A precursor is suitable for CVD if it is sufficiently volatile, chemically pure, stable during evaporation, cost-effective, and nonhazardous and has prolonged shelf life.^24^ Additionally, its decomposition did not leave residual impurities. For example, in carbon nanotube fabrication via CVD, a substrate in an oven undergoes high temperatures, and a carbon-containing gas (such as hydrocarbons) is gradually introduced as a precursor. At elevated temperatures, the gas decomposes, releasing carbon atoms that recombine to form carbon nanotubes on the substrate.^24^ The catalyst selection significantly influences the morphology and type of nanomaterial produced. In CVD-based graphene synthesis, Ni and Co catalysts yield multilayer graphene, whereas a Cu catalyst yields monolayer graphene. Overall, CVD is exceptional for generating high-quality nanomaterials and is renowned for its effectiveness in producing two-dimensional nanomaterials.^24^

### 3.3 Biological Methods

#### 3.3.1 Green Synthesis

The nanotech industry promotes the use of nano as an eco-friendly solution to enhance the environmental impact of existing industries.^25^ It targets reduced resource and energy consumption for sustainable economic growth. Eco-conscious approaches, especially plant-extract-mediated nanoparticle synthesis, stand out compared to microorganisms owing to the shorter cell maintenance time.^26^ The essential steps include preparing leaf extracts, phytochemical screening, and precursor preparation for nanoparticle synthesis and characterization. Factors such as pH, temperature, and time influence synthesis. The green approach, which utilizes plant extracts, degrades organic compounds, mainly polyphenols. Although nanoparticle synthesis has increased, their limited use in wastewater treatment is changing, offering a potential alternative water source.^27^

### 3.4 Nanotechnology and Various Nanomaterial Applications

There are four primary categories of nanomaterials: carbonaceous, metallic, dendritic, and composite nanomaterials.^28^ Since ancient times, carbon materials have played a pivotal role in shaping human life, finding widespread use in households, and meeting day-to-day needs.^29^ The advent of nanotechnology has further ushered in various nanoforms of carbon materials that operate at the molecular and submolecular levels. These carbon nanomaterials exhibit distinct properties compared to their bulk-scale counterparts, sparking significant interest among researchers who have delved into their electrical, physical, mechanical, sensing, and chemical attributes.^29^ The current state of research is on the diverse allotropes of carbon nanomaterials and their inherent properties. There is an imperative need for functionalization of carbon materials, which is essential for a spectrum of applications. The growing commercial utilization of these materials spans the technical, environmental, and agricultural domains.

### 3.5 Dendrimers

Dendrimers exhibit a highly branched molecular structure, characterized by intricate 3D branching.^30^ These branches have generated significant enthusiasm for the attachment of diverse molecules, enhancing properties such as solubility and bioavailability. Applications of dendrimers are primarily concentrated in various drug delivery domains, including gene delivery, controlled drug release, and antimicrobial and anticancer therapies.^30^ Biosensors based on dendrimers are predominantly crafted through layer-by-layer assembly and serve as glucose-sensing devices, electrochemical detectors, fluorescence detectors, and quartz crystal microbalance (QCM) detectors.^31^

### 3.6 Composite Nanomaterial

Natural polymer nanocomposites have gained attention in scientific and industrial circles, addressing the environmental concerns linked to petroleum-based polymers.^32^ Comprising biopolymers such as chitosan, starch, cellulose, and alginate from diverse sources, eco-friendly nanocomposites are useful in agriculture and food.^33^ Acting as slow-release nanocarriers, they enhance crop yield by delivering agrochemicals. Biopolymer-based nanofilms and hydrogels serve as coatings, prolong shelf life, aid seed germination, and safeguard against pathogens. In food packaging, blending biopolymers with nanofillers improves the mechanical strength and barriers. This article outlines the applications of nanocarriers, hydrogels, and coatings in food and agriculture. Despite its potential benefits, it also delves into the risks, challenges, opportunities, and consumer perceptions tied to nanotechnology in agriculture, food production, and packaging.^33^ These insights will contribute to the continued evolution of nanotechnology and its integration into various scientific and industrial domains.

## 4 Nanotechnology Approaches for Remediation of Soil Pollution

Nanotechnology is an emerging paradigm in agriculture, particularly for enhancing plant phytoremediation capabilities for soil and water, indicating its potential in the agricultural sector.^34^ Nanoparticles offer distinct advantages over traditional soil remediation methods, primarily because of their size and surface area.^35^ This small particle size enables remarkable efficacy in nanotechnology applications. This explores the utilization of nanotechnology in phytoremediation and explores its applications and future prospects. Various types of nanoparticles are effective in cleansing and detoxifying diverse pollutants, as discussed comprehensively herein. Ongoing research and interdisciplinary collaboration promise further research in this intriguing field. Although planetary reclamation remains challenging, nanophytoremediation has emerged as a promising solution to address these environmental issues. Soil pollution and degradation pose urgent global environmental challenges that impact agricultural productivity, food security, and human well-being.^36^ The depletion of soil resources owing to escalating food production demands for the growing human population has contributed to widespread soil exploitation and deterioration.^36^ Additionally, soil contamination with heavy metals, pesticides, and persistent organic pollutants (POPs) has intensified this crisis. Polluted soil containing these substances increases the risk of contaminating the food chain through the bioaccumulation of pollutants.^37^

**Figure 3:**
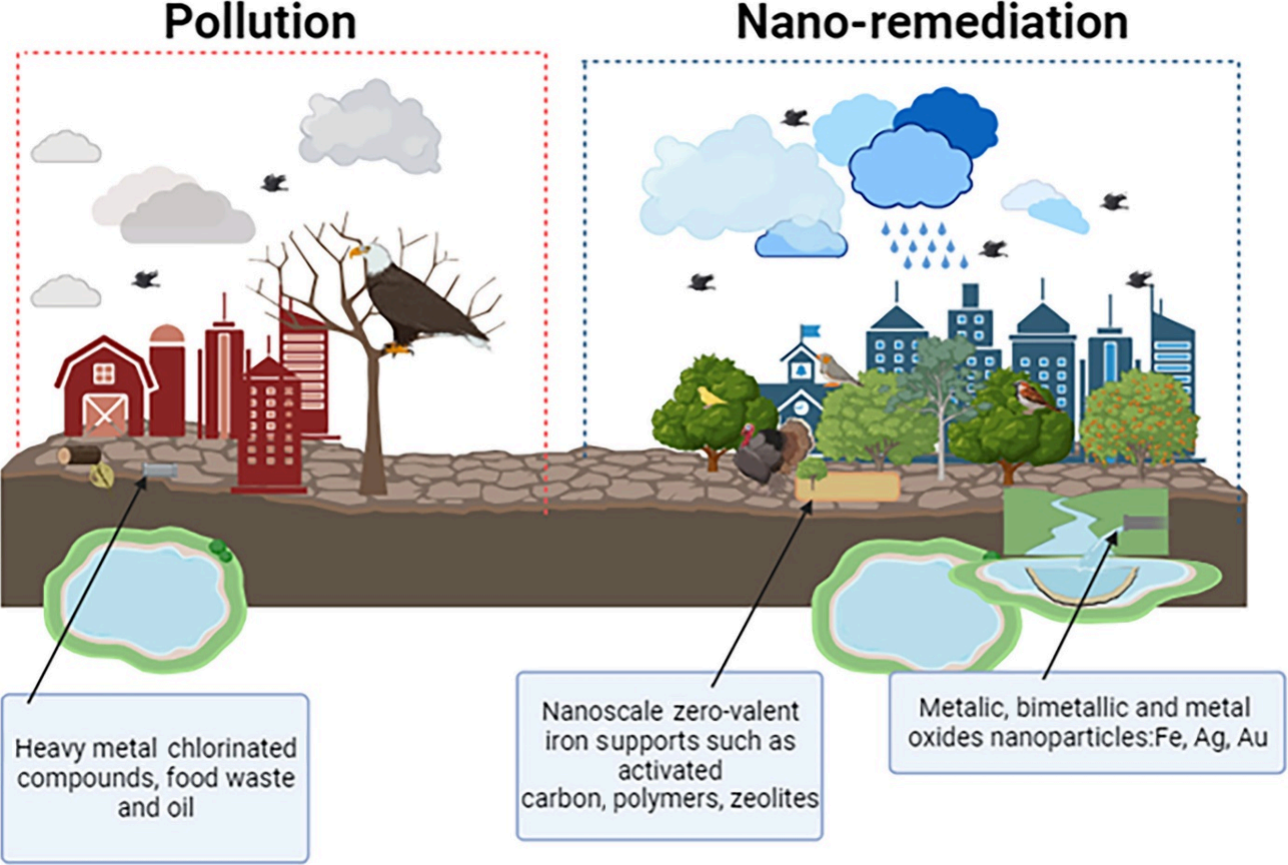
Environmental pollution remediation using nanotechnological approaches.

The concurrent challenges of meeting the increased food production needs and preventing further soil degradation severely hamper agricultural productivity. Nanoenabled soil remediation has emerged as a promising and sustainable solution to revitalize compromised soil resources.^38^ Nanotechnology applications are cost-effective and user-friendly and offer efficient treatment and remediation approaches to significantly mitigate soil pollution.^39^ This explores the potential of nanotechnology-based soil remediation, specifically addressing heavy metals, pesticides, their residues, and POPs while also examining their role in enhancing phytoremediation and bioremediation.^40^ Thus, the global focus on nanotechnology for soil remediation has intensified. This study explores contaminant fate in the soil, detailing nanotechnology mechanisms with various nanomaterials for remediation. It assesses the pros and cons of nanomaterials for terrestrial organisms, human health, and soil. Challenges in nanotechnology for soil remediation have been highlighted, with a significant concern being the adverse impact of nanoparticles on microbes, potentially inhibiting enzyme functions in the soil (Figure 3).

Numerous applications of nanotechnology exist, and there is ample evidence of the new uses of nanoremediation, particularly with regard to soil pollution. Iron nanoparticles have an exceptional 100% removal effectiveness for hexavalent chromium.^41^ Nanomaterials in soil remediation reduce pollutants, cleanup time, and costs and eliminate soil disposal.^42^ nZVI nanoparticles immobilize heavy metals, whereas carbon nanotubes offer a high adsorption capacity for organic and inorganic cleanup.^43^ Recently, studies on the biological production of nZVI soil remediation materials have also been conducted, with promising outcomes.^43^ Even though nanoparticles contribute to soil remediation, the increasing accumulation of metal and metallic oxide engineered nanoparticles (ENPs) in agricultural soils poses a significant threat to ecosystems and soil health.^44^ These nanoparticles alter the pH, conductivity, redox potential, and soil organic matter content, increase hydraulic conductivity, and interact with nutrients, thereby reducing their bioavailability.^44^ Soil quality and health are significantly influenced by chemical and physical characteristics, which may decline owing to the annual influx of ENPs.^44^ Additionally, nutrients, ENPs, or cations released in soil can interact to generate complexes or precipitates that alter the availability of nutrients in the soil solution.^44^ Although previous studies have established the efficacy of nanotechnology in soil remediation, newer dimensions have focused on customization, sustainability, smart delivery systems, interdisciplinary collaborations, and a comprehensive understanding of potential risks. These insights contribute to the continued advancement of nanotechnology for sustainable and effective remediation of soil pollution.

## 5 Mechanism of Nanotechnology: Reduction Reaction, Immobilization, Nanobiosorbents, and Nanobiosurfactants

*In situ* techniques are widely employed in soil remediation. Various technologies, such as adsorption, immobilization, Fenton and Fenton-like oxidation, reduction reactions, and combinations of nanotechnology and bioremediation, have been utilized for the remediation of soil contaminants.^45^ The synergistic mechanism of combining nanotechnology and bioremediation has recently raised significant concern. A summary of nanomaterials and nanotechnology applied for the *in situ* removal of contaminants from soils, including heavy metals, organic compounds, and metalloids, is presented in Table 1. Inorganic contaminants, such as heavy metals and metalloids, are typically eliminated through adsorption facilitated by nanoparticles. Simultaneously, organic contaminants are removed via reduction reactions and degradation in the presence of catalysts. The use of nanomaterials enhances the processes of adsorption and oxidation, enabling the degradation and removal of micropollutants that persist in the soil environment.^46^ Widely used nanotechnological applications in soil remediation for contaminant removal include carbon nanomaterials, iron(III) oxide (Fe_3_O_4_), titanium oxide (TiO_2_), zinc oxide (ZnO), nZVI, and nanocomposites.^47^ Notably, nZVI is the most commonly used nanoparticle for eliminating heavy metal pollutants owing to its high efficiency in transforming contaminants such as toxic metals, chlorinated organic compounds, and inorganic compounds into less harmful forms.^48^

**Table 1:** List of Heavy Metals and Environmental Pollution Sources

S. No.	Heavy metals	Sources	Ref
1	Cr(VI)	Ferroalloys, mining, the leather industry, and metallurgy, etc.	(69)
2	Pb^2+^	Pesticides, fertilizers, batteries, metal plating, and ore smelting	(70, 71)
3	As	Coals, ceramics, metallurgy, animal supplements, electrical production, geochemistry, and pesticides.	(72)
4	Cd^2+^	Coal burning, pigments, and metal coating batteries	(73)
5	Hg^1+^	Among these are the industries of metallurgy, catalyst, mercury lamps, paper and pulp, pharmaceuticals, and agriculture	(74)
6	Ni^2+^	Glass batteries, ceramics, and catalyst	(75, 76)
7	Cu^2+^	Water pipelines, metals, and the chemical and pharmaceutical sectors	(75, 76)
8	Zn^2+^	Rubber, paint, PVC stabilizers, zinc alloys, and stabilizers	(77)

### 5.1 Reduction Reaction

Reduction reactions, facilitated by nZVI nanoparticles, exhibit significant potential for eliminating heavy metals and organic compounds from contaminated soil as well as addressing water and groundwater contamination.^49^ The widespread application of nZVI particles in various fields is attributed to their nanoparticle size and large surface area, which enhance remediation efficiency by direct contact with contaminants. The injection of nZVI particles into contaminated soil demonstrates their strong reduction capacity and effective adsorption ability, transforming toxic contaminants such as chromium(VI) into less harmful compounds such as chromium(III), and the formation of new compounds such as ferrous chromite.^43^ The addition of biochar to nZVI nanoparticles enhances the reduction reaction capacity and removal efficiency, reinforcing iron particle disparity and reducing mixture movement in the soil.^50^ For example, the combined use of biochar and nZVI removed 66% of the Cr(VI) content in the soil. In one investigation, 28% of 1 kg of chromium(VI) was reduced with 1 g of nZVI injected into contaminated soil. Under conditions with a pH of 5, 98% of chromium(VI) was removed within 24 h.^50^

### 5.2 Immobilization

The *in situ* immobilization mechanism for contaminants has gained significant global attention as it represents a cost-effective and environmentally friendly approach for remediating contaminated soil.^51^ The selection of nanomaterials for immobilization remediation depends heavily on contaminant properties and soil conditions. Commonly employed nanoparticles for immobilization remediation include carbon and metal oxide nanomaterials.^52^ Carbon nanomaterials, such as fullerene, carbon nanotubes, and graphene, act as adsorbents in immobilization remediation, utilizing van der Waals forces and π–π interactions to absorb organic contaminants.^53^ The hydrophobic surface characteristics and high adsorption ability of carbon nanomaterials enhance their efficacy in removing organic contaminants from soil. Carbon nanotubes, in particular, exhibit high adsorption properties for organic compounds compared to organic matter in soils. They display specific adsorption toward ionizable organic compounds, such as pesticides, through π–π and cation−π interactions. In addition, carbon nanotubes have a low-barrier surface and form hydrogen bonds with electron charges. The application of carbon nanotubes has been extensively studied under various conditions to reduce organic compounds such as polycyclic aromatic hydrocarbons (PAHs).^54^ For example, the presence of carbon nanotubes in soil impedes the movement of PAHs, thereby reducing their bioavailability to crops and microorganisms in the soil environment. The oxygen content influences the adsorption capacity of carbon nanotubes, with the −OH functional group enhancing the adsorption capacity by strengthening the interactions between π and π and −OH.^55^

### 5.3 Nanobiosorbents

The revolutionary development of the industrial sector and urban orientation in present times has heightened global pollution. Environmental evaluation shows the presence of multiple contaminants in the environment, which ultimately leads to hazardous impacts on the lives of humans, animals, and plants accompanying the loss of aesthetics. This critical issue has led scientists and researchers to develop environmentally friendly, economically affordable, and promising techniques for the removal of contaminants. One such approach is the development of nanobiosorbents for contaminant removal using multiple renewable and natural sources.^56^ Nanosorbent materials are widely regarded as the most effective approach for remediating water and wastewater because of their broad applicability and abundance of available adsorbents.^57^ An extensive variety of biosorbents and nanoadsorbents exist for the purpose of eliminating impurities from water.^58^ These include microbial biomass, agricultural wastes, nano-MgO, Fe_3_O_4_ nanoparticles, CaO/Fe_3_O_4_ nanoparticles, and activated carbon/Fe_3_O_4_ nanocomposites, which is a composite of nanoadsorbents and biosorbents.^59^

### 5.4 Nanobiosurfactants

The widespread global pollution of coastal regions has led to the contamination of marine sediments, particularly by persistent pollutants, such as PAHs, crude oil, halogenated compounds, and metals, posing significant public health and environmental concerns. These contaminants affect the well-being of populations, marine ecosystems, fisheries, and overall economic landscape. To sustainably address this issue, there is a critical need for eco-friendly solutions for the remediation of polluted marine sediments. Although physiochemical methods are robust, microbial/plant-based biological remediation approaches are gaining preference, despite challenges in solubilizing certain pollutants. This has led to the increased exploration of consolidated biotechnologies involving biosurfactant supplementation in remediation systems. Biosurfactants, comprising amphipathic biomolecules, offer unique properties, such as surface tension reduction, high emulsification, wettability, low critical micelle concentration, increased solubility, low toxicity, and chemical stability under extreme environmental conditions.^60^ This section delves into the role of biosurfactants in remediating organically contaminated marine sediments, focusing on the environmental sustainability of various coastal areas. The discussion includes biosurfactant production under aerobic and anaerobic conditions, environmental suitability properties, application strategies, and interaction mechanisms between biosurfactants and pollutants during remediation. Recent advances and future prospects for developing efficient and eco-sustainable biosurfactant-based strategies for marine sediment remediation are also presented.^61^ We conclude that while previous studies have laid the groundwork for understanding the mechanisms of nanotechnology in reduction reactions, immobilization, nanobiosorbents, and nanobiosurfactants, newer dimensions emphasize green synthesis, improved immobilization strategies, expanded applications of nanobiosorbents and nanobiosurfactants, and interdisciplinary collaboration. These insights will contribute to advancing the efficiency, sustainability, and broader applicability of nanotechnology in diverse fields.

## 6 Remediation of Heavy Metals Using Nanotechnology

Heavy metal contamination is a major environmental issue worldwide. The gradual increase in heavy metal contamination of soil due to human activities such as mining and urbanization is one of the most important causes of concern. Large volumes of garbage are produced during mining activities and are gathered at waste accumulation sites.^62^ These expanding trash heaps have a negative effect on some places and may turn some agricultural regions into wastelands.^63^ When exposed to concentrations exceeding the recommended limits, heavy metals cause harmful toxicity to aquatic organisms, plants, and humans. Heavy metals are highly toxic. Although most people associate heavy metals with toxicity to living things, lightweight metals, such as beryllium and lithium, can also be harmful. Certain heavy metals, such as Cr^3+^, Fe^3+^, Fe^2+^, and so forth, are necessary for human health and are safe in moderation. The degree of metal toxicity is determined by the exposure route, duration, and dose/quantity, all of which can lead to acute or chronic toxicity. Although chromium (Cr) exists in a variety of oxidation states, the most stable forms are ^+3^ and ^+6^. Humans require chromium in its ^+3^ form because of its unique nutritional and biological properties.^64^ Heavy metals are naturally occurring, but they are being produced and released into the environment at an alarming rate due to increased industrialization and urbanization (Table 1). Nowadays, the use of biosynthetic nanoparticles in nanotechnology is a suitable approach to remove contaminants from the atmosphere. Adsorption is a common approach used in heavy metal removal. Because of their minuscule size and large surface area, nanomaterials are effective sorbents with enormous adsorption capabilities that may remove heavy metal ions from contaminated water.^65^

Nanomaterials are sufficiently small to alleviate some of the problems associated with traditional site rehabilitation at a reasonable cost. They could also be suspended for sufficiently long periods to start the creation of an *in situ* target.^66^ Nanoremediation has the same *in situ* and *ex situ* capabilities as conventional techniques^67^ (Table 2). In the *in situ* technique, contaminants are remedied at the source. In the *ex situ* approach, they are transferred to another location for remediation.^67^ Data from numerous studies suggest that the use of nanoparticles could improve the phytoremediation of Pb, Cr, Cd, Zn, and Ni.^38^ According to recent research, metal oxide nanoparticles are promising for eliminating hazardous metal ions from wastewater.^68^ Because metallic nanoparticles are unstable when they agglomerate or separate, only a small number of them have been examined for sorption. Moreover, separating individual metallic nanoparticles from the effluent is a challenging procedure. However, earlier research has demonstrated the potential of nanotechnology in remediating heavy-metal pollution; newer dimensions emphasize tailored nanomaterial properties, green synthesis approaches, multifunctional materials, *in situ* applications, and advanced monitoring techniques. These insights contribute to the ongoing evolution of nanotechnology for sustainable and effective remediation of heavy-metal-contaminated environments.

**Table 2:** Different Nanoparticles Were Used for Bioremediation

S. No.	Nanoparticles	Application/advantage	Ref
1	Silica nanoparticles	Photocatalytic degradation	(78)
2	Graphene oxide and carbon nanotubes	High surface area, the presence of pores between MOFs and platforms, hydrophobic and/or π–π interactions, and a variety of morphological characteristics of mixed nanocomposites	(79)
3	Enzyme immobilized nanoparticles	Immobilized laccase oxidation	(80)
4	Zirconia nanoparticles	Strong electrostatic interactions and chemisorptions between zwitter ions	(81)
5	Electrospun cyclodextrin fibers	Bacterial bioremediation	(82)
6	Mesoporous organosilica nanoparticles (MONs)	More surface area and conjugation resulting from ferrocene-mediated noncovalent interaction	(83)
7	Cobalt and cobalt oxide nanoparticles	Sunlight and a huge surface area	(84)
8	NiO and MgO nanoparticles	Zn^2+^ adsorption is exothermic and chemical, while spontaneous, endothermic, and physical adsorption of Cu^2+^ and Cr^3+^	(85)
9	Electrospun nanofibrous webs	Biological elimination of color	(86)

### 6.1 Nanobioremediation of Organic Contaminants in Soil

Environmental contaminants, such as heavy metals and organic and inorganic pollutants, can be eliminated from contaminated areas by employing nanoparticles or nanomaterials made by plants or microorganisms, such as bacteria or fungi, and nanotechnology.^87^ This process is known as nanobioremediation. Nanobioremediation has gained acceptance as a versatile tool for long-term environmental restoration.^87^ According to recent developments, bioremediation currently offers an economically viable and environmentally beneficial way to remove contaminants from the environment.^88^ The three main bioremediation techniques are microbial, plant, and enzyme-mediated remediation. One such technique that uses biological and physiochemical methods is nanobioremediation, which is currently being studied in a number of polluted locations. In the nanobioremediation process, pollutants are broken down to a level that is suitable for biodegradation using nanomaterials, and subsequently, the contaminants undergo biodegradation.^87^ Nanoparticles generated biologically from microbes or plant extracts are used in nanobioremediation to remove pollutants from land and water. Over the past 20 years, nanomaterials have emerged as strong contenders to replace traditional therapeutic approaches because of their high efficacy, affordability, and environmental friendliness.^88^

Numerous viable iron-based treatments are available for the cleanup of contaminated soil and groundwater.^89^ By solubilizing heavy metal contaminants at their interface, zerovalent iron NPs have been shown to effectively remediate acidic water contaminated with heavy metals, making them a practical and essential method of nanoremediation.^90^ Biologically produced nanoparticles are used in nanobioremediation, a cutting-edge and rapidly developing novel technique to remove pollutants from the environment.^91^ In an effort to increase crop yields, the use of nanomaterials in agriculture, such as nanopesticides, nanofertilizers, and sensors, is receiving more attention. Many researchers have investigated the potential of nanotechnology, namely, the method of nanoencapsulation for pesticide dispersion.^92^ As a naturally occurring method of crop protection, the creation of a nanoencapsulated pesticide can reduce the use of pesticides and, consequently, human contact. Pesticide degradation is significantly influenced by the unique and explicit surface area behaviors of nanomaterials.^93^ Although previous studies have established the potential of nanobioremediation for organic contaminants in soil, newer dimensions emphasize precision, sustainability, synergy with microbial communities, smart delivery systems, biodegradability, field-scale applications, and advanced monitoring techniques. These insights contribute to the ongoing development of robust and environmentally friendly nanobioremediation strategies for organic soil contaminants.

### 6.2 Nanophytoremediation of Soil Pollutants

The potential toxicity of heavy metals makes soil poisoning a major global concern. Soil pollution by heavy metals poses significant risks to human health and ecology.^94^ Heavy metal contamination poses a significant risk to the environment and food security owing to the rapid expansion of the agricultural sector and related industries.^63^ In addition, the massive expansion of the global population has led to a disturbance in the natural habitat, which has raised the level of heavy metal contamination on Earth. One of the more important fields of environmental research is the management and prevention of heavy-metal contamination.^63^ Chemical, physical, and biological methods have been used to extract heavy metals from the environment. The soil microbial ecosystem is destroyed, and physical and chemical rehabilitation methods are expensive and have a negative impact on the soil texture.^88^

Toxins are eliminated by bioremediation, which uses a variety of techniques, including bacteria, plants, and animals, without damaging the environment.^38^ Phytoremediation is a successful, eco-friendly, and reasonably priced type of bioremediation.^38^ This technique is increasingly being used to clean areas contaminated with toxic organic compounds and heavy metals.^38^ Additionally, radioactive pollutants can be eliminated from agricultural fields and groundwater using this technique. A cheap technique known as phytoremediation functions best when pollutants are found in the root zones of plants. Because flax (*Linum usitatissimum*) can be grown to generate flax seeds and can remove large amounts of Cu from soils, it is a suitable candidate for the phytoremediation of Cu.^95^ Several phytoremediation procedures, such as phytostabilization, rhizofiltration, phytoextraction, and phytovolatilization, can be used to remove heavy metal contaminants.^96^ Rhizospheric bacteria, in addition to plants, play a critical role in the process of cleaning up contaminated environments. The same principles of phytoremediation that nature utilizes are employed by microorganisms and plants to decrease organic and inorganic contaminants.^97^ While previous studies have demonstrated the potential of nanophytoremediation of soil pollutants,^98^ newer dimensions emphasize a deeper understanding of uptake mechanisms, enhanced plant–microbe interactions, tailored nanoparticle design, multicontaminant remediation, green synthesis, field-scale applications, risk assessment, and community engagement. These insights will contribute to the ongoing development of effective and sustainable nanophytoremediation strategies for diverse soil pollution challenges.

### 6.3 Nanotechnology in Agricultural Soil Remediation: Innovations and Sustainable Practices

The remediation of polluted agricultural soils has emerged as a critical area of research, and nanotechnology offers innovative approaches to address this pressing environmental concern. In recent years, a myriad of nanomaterials have been explored for their potential in soil remediation, highlighting the interdisciplinary nature of nanotechnology and environmental science.^6^ One notable avenue of research involves the use of nZVI particles, which exhibit excellent reactivity in the degradation of various pollutants.^99^ These particles can be tailored to specific contaminants and offer a promising means of enhancing the soil quality. Additionally, the application of nanomaterials, such as TiO_2_ and carbon-based nanoparticles, has shown remarkable effectiveness in adsorbing and transforming pollutants, mitigating the adverse effects of agricultural activities on soil health.^100^ Furthermore, nanoscale delivery systems, including nanocarriers and nanosensors, have paved the way for precision agriculture for pollutant management.^101^ These nanodevices enable the targeted delivery of remediation agents and real-time monitoring of soil conditions, providing a proactive and sustainable approach to agricultural soil remediation. Despite these promising prospects, it is essential to carefully assess the potential risks associated with the deployment of nanomaterials in agricultural settings. Understanding the fate, transport, and toxicity of nanoparticles is crucial for ensuring the long-term sustainability and safety of nanotechnology-based soil remediation strategies. The integration of nanotechnology into agricultural soil remediation holds great promise, offering novel and efficient solutions to the complex challenges posed by soil pollution. This review aims to synthesize recent advancements in nanotechnology for agricultural soil remediation, highlighting both the opportunities and challenges that lie ahead for sustainable and environmentally friendly practices in modern agriculture.

### 6.4 Challenges and Future Directions of Nanotechnology for the Remediation of Soil Pollution

The presence of NPs in soils is reported to alter soil pH, which is one of the most important parameters that influences soil nutrient availability, microbial dynamics, overall soil health, and plant growth and development.^102^ It has been shown that the presence of NPs in soils changes the pH, one of the key factors influencing soil nutrient availability, microbial dynamics, general soil health, and plant growth and development.^102^ Additionally, it has been noted that NPs of Ag, Au, Ti, and Zn alter soil pH and that their presence has a negative impact on nematodes and beneficial soil microbes.^103^ The type and concentration of NPs present in the soil, the type of soil, and the enzymatic activity of the soil all affect the extent of their adverse effects. Furthermore, a decrease in dehydrogenase activity is linked to an increased NP concentration, which increases the balance between soil fertility and nutrient levels. Additionally, the uptake and assimilation of these nanoparticles by microorganisms profoundly affect the mycelium, impairing their regular cellular operations.

In this study, we conducted a comprehensive review of the use of nanotechnology in agricultural settings for soil bioremediation to mitigate the impact of pollutants. We extensively examined the existing literature, specifically focusing on review articles. Following this thorough review, we formulated a detailed methodology section outlining the procedures and approaches employed in the analysis. The methodology encompasses our systematic exploration of the use of nanotechnology for the bioremediation of agricultural soils contaminated with pollutants, synthesizing information obtained from relevant review articles. By combining biotechnology with nanotechnology, enzymes enclosed in nanoparticles convert complex organic compounds into simpler ones that are swiftly removed by bacteria and plants. In addition to vascular plants, microorganisms, such as bacteria, filamentous fungi, yeasts, algae, and actinobacteria, can be used to synthesize biogenic nanoparticles. Because iron oxide and magnesium oxide NPs have smaller sizes and fewer interactions with their surroundings, they decrease the bulk density of agricultural soils by 8% and 11%, respectively.^100^ The aggregation of sandy loam soil was enhanced by 35% by carbon nanotubes because of their exceptional elastic capabilities and high aspect ratios. NPs can be used in various ways to enhance the hydrological regimes of soils. For example, they can be used to build water-absorbing hydrogels or to enhance the surface area and hydrophilicity of soil particles to boost the ability of the soil to store water. This can assist plants to adjust to water scarcity and drought stress.^100^ Zeolites, silica, chitosan, alginate, and polymers are some of the most frequently utilized nanofertilizers, which function as slow-releasing fertilizers, reduce environmental losses, and boost nutrient efficiency.^100^

## 7 Conclusions

The incorporation of nanotechnology into the process of remediating soil contamination signifies a paradigm shift. This study’s investigation of diverse methodologies and nanomaterials highlights the potential for inventive and environmentally sound resolutions. Nanotechnology not only provides solutions for the pressing issue of soil pollution but also presents opportunities for environmental health in the future. It is crucial to maintain the momentum of research and development regarding the implementation of nanotechnology to guarantee its safety and effectiveness across a wide range of soil conditions. Continuous advancements in nanotechnology are pivotal for future remediation of soil contamination. To improve the efficacy and security of nanomaterials, exhaustive investigation of their enduring ecological ramifications is imperative. Furthermore, it is imperative to investigate uncharted territories, including the advancement of innovative nanomaterials and the refinement of nanophytoremediation methodologies. Effective cooperation among scientists, policymakers, and industry stakeholders is critical for successful integration of nanotechnology into soil remediation processes. The ongoing evaluation and monitoring of the performance of nanomaterials under various soil types and conditions will aid in the formulation of effective remediation strategies tailored to specific sites. A forward-thinking perspective necessitates the integration of sustainable practices with nanotechnology to safeguard and restore soil health for future generations.
